# Bioinformatics Analysis Reveals the Oncogenic Role and Therapeutic Potential of lncRNA SNHG25 in Colon Adenocarcinoma

**DOI:** 10.1155/ijog/4528082

**Published:** 2025-08-23

**Authors:** Renshan Hao, Ye Zhang, Qi Zhu, Pufang Tan

**Affiliations:** ^1^Division of Gastroenterology and Hepatology, Baoshan Branch, Renji Hospital, Shanghai Jiao Tong University School of Medicine, Shanghai, China; ^2^Laboratory of Medicine, Baoshan Branch, Renji Hospital, Shanghai Jiao Tong University School of Medicine, Shanghai, China

**Keywords:** colon adenocarcinoma, long noncoding RNA, predictive drug, SNHG25

## Abstract

**Background:** Colon adenocarcinoma (COAD) is a common digestive malignancy with limited therapies and a poor prognosis. Previous studies have highlighted lncRNAs' key role in cancer, but the exact function of lncRNA *SNHG25* in COAD remains unclear.

**Methods:** In this study, we obtained COAD transcriptome data from the UCSC Xena database, screened for differentially expressed genes, and assessed the diagnostic efficacy of *SNHG25* using the DESeq2 package. Subsequently, the *SNHG25* high- and low-expression groups were enriched and analyzed for immune cell infiltration characteristics and correlation with *SNHG25* using the CIBERSORT and ESTIMATE algorithms. Its impact on immunotherapy and drug sensitivity was assessed by combining TIDE with oncoPredict database. The target mRNAs were further screened by Encori platform and potential target drugs were predicted using molecular docking technology. Finally, qRT-PCR, CCK-8, wound healing, and transwell assays were used to assess the mRNA expression levels and potential biological functions of *SNHG25*.

**Results:** *SNHG25* expression level was upregulated in COAD samples, and the ROC curve showed the area under curve (AUC) value = 0.937, revealing its strong diagnostic ability. Functional enrichment analysis showed that its high expression was associated with activation of oxidative phosphorylation pathway, while low expression was enriched in apoptosis and immune-related signaling pathways. Immune infiltration analysis showed that *SNHG25* was significantly associated with a variety of immune cell subtypes (e.g., macrophages and neutrophils) and might be involved in the remodeling of the tumor immune microenvironment. *ZMYND8* was identified as a key downstream mRNA target (AUC = 0.811), and three potential therapeutic drugs—demecolcine, piroxicam, and vorinostat—were predicted based on DSigDB screening and validated by molecular docking, with binding energies of −6.48, −7.15, and −5.39 kcal/mol, respectively. Finally, in vitro cellular assays confirmed that *SNHG25* expression was elevated in COAD cell lines (*p* < 0.0001), and its knockdown significantly suppressed cell proliferation, migration, and invasion.

**Conclusion:** This study highlights that *SNHG25* is highly expressed in COAD and promotes tumor progression through multiple mechanisms, advancing research and treatment strategies for this malignancy.

## 1. Introduction

Colorectal cancer, encompassing cancers of the colon and/or rectum, ranks among the most prevalent cancers in both men and women. It is the third most commonly diagnosed cancer type, accounting for 11% of all cancer diagnoses, and it stands as the second foremost cause of cancer-related deaths globally [1, 2]. Colon cancer deaths are projected to increase by 71.5% by 2035, highlighting a growing public health concern [3, 4]. Colon adenocarcinoma (COAD) is the most common type of colon cancer. Currently, surgery is the primary treatment for COAD, mainly targeting patients in early and intermediate stages, while chemotherapy and radiotherapy are the mainstays for advanced patients [5, 6]. Notably, the early symptoms of COAD are often inconspicuous, with significant symptoms typically emerging only in later stages, significantly limiting opportunities for timely intervention and treatment [7]. The development of targeted therapy and immunotherapy has offered new possibilities for the treatment of COAD, but there are still gaps in the research on corresponding predictive biomarkers and targets. Crucially, despite relentless efforts by the scientific community, groundbreaking therapeutic drugs for COAD remain scarce, struggling to meet the escalating clinical demands and patients' aspirations for improved quality of life.

Long noncoding RNAs (lncRNAs) refer to the noncoding transcripts with a length exceeding 200 base pairs [8, 9]. lncRNAs are deeply involved in core biological processes, including cell proliferation, survival, migration, and genomic stability [10, 11]. In addition, their abnormal expression is involved in a variety of human diseases, particularly almost all types of cancers [12]. For example, studies have shown that lncRNA *SCARNA2* is overexpressed in tumor tissues in COAD [13]. Moreover, overexpressed lncRNA *SNHG1* could promote epithelial–mesenchymal transition (EMT) through binding to *miR-497*/*miR-195-5p* in COAD cells [14]. Building on these findings, *SNHG25*, an emerging member of the lncRNA family, has attracted increasing attention. Previous studies have reported its upregulation in various tumors, where its knockdown significantly suppresses cancer cell proliferation, migration, and invasion, suggesting a potential oncogenic role. Specifically in COAD, low expression of *SNHG25* has been closely associated with reduced metastatic potential, indicating its pathological relevance in this cancer type [7]. However, the regulatory mechanisms of *SNHG25* in COAD remain largely unexplored. Its involvement in immune-related features and therapeutic targeting has yet to be fully elucidated. Thus, a comprehensive investigation into the molecular functions of *SNHG25* in COAD is of great theoretical and clinical significance.

Given the current limited understanding of the function of lncRNA *SNHG25* in COAD and its involvement in drug regulation pathways, this study attempts to analyze the molecular mechanism of *SNHG25* in COAD through differential analysis, enrichment analysis, immune infiltration analysis, and other methods. Furthermore, we aim to leverage relevant mRNAs for drug prediction and molecular docking, thereby offering fresh perspectives for advancing COAD research and drug development.

## 2. Materials and Methods

### 2.1. Data Acquisition and Preprocessing

The count and FPKM data of the COAD dataset were collected from the UCSC Xena database (https://xena.ucsc.edu/). After screening and retaining the 01A cancer samples and 11A adjacent cancer samples labeled as adenocarcinoma or adenoma, a total of 424 samples were finally obtained, including 385 cancer samples and 39 adjacent cancer samples. In addition, we obtained the GSE146009 dataset (65 samples) from the Gene Expression Omnibus (GEO) database (https://www.ncbi.nlm.nih.gov/), including 33 tumor tissue samples and 32 control samples, which was used to revalidate the expression level of *SNHG25*.

In addition, we retrieve 580 apoptosis-related genes from prior research to assemble a gene set linked to apoptosis [15].

### 2.2. Screening and Analysis of Differentially Expressed Genes (DEGs)

To distinguish between tumor and adjacent noncancerous control groups, we used the DESeq2 package to perform differential expression analysis based on raw, unnormalized count data. DESeq2 applies a negative binomial distribution model and adjusts for multiple testing using the Benjamini–Hochberg method [16]. Genes with an absolute log2 fold change (|log2FC|) ≥ 1 and adjusted *p* value < 0.01 were considered significantly differentially expressed [17]. Subsequently, the Top 20 genes with the most significant high and low expressions were selected, along with our gene of interest, *SNHG25*, to jointly plot a heat map of their expression levels using pheatmap package [18]. Furthermore, to assess the prediction capability of the *SNHG25* gene in distinguishing between tumor and normal tissues, we will generate a receiver operating characteristic (ROC) curve.

### 2.3. Enrichment Analysis

To examine the potential role of *SNHG25* in tumor apoptosis, single sample GSEA (ssGSEA) was performed using apoptosis-related genes from previous studies [15] as the background gene set to calculate the programmed death scores (PDSs). Following this, we performed a Spearman correlation analysis between the expressions of *SNHG25* and these apoptosis-related scores.

To understand the differences in relevant biological functions and involved signaling pathways between the high- and low-expression groups of *SNHG25*, the samples were divided by the median gene expression into low- and high-expression groups. Enrichment analysis was then performed in the background gene set using GSEA_4.2.2 software [19], with a significance threshold of false discovery rate (FDR) < 0.05.

### 2.4. Immune Infiltration Analysis

Using the CIBERSORT package [20] in R, we estimated the relative proportions of immune cell infiltration in COAD and control samples. The analysis was performed based on the LM22 signature matrix, which defines 22 functionally distinct human immune cell subsets, including various types of T cells, B cells, natural killer (NK) cells, macrophages, dendritic cells, and others. LM22 was selected because it is one of the most widely validated immune deconvolution references for bulk transcriptomic data, particularly in tumor-related studies. To further explore the immunological relevance of *SNHG25*, we also calculated the correlation between its expression and immune cell infiltration using the Spearman algorithm. We utilized the estimate package [21] in R to conduct immune infiltration analysis. Furthermore, we calculated the Spearman correlation coefficient between *SNHG25* and the ESTIMATE score, immune score, and stromal score [22].

### 2.5. Immunotherapy Correlation and Drug Sensitivity Analysis

Using the TIDE (http://tide.dfci.harvard.edu/) database, we assessed the potential clinical efficacy of immunotherapy in dataset, evaluating the correlation between specific genes and the likelihood of benefiting from immunotherapy. The samples in the dataset were analyzed using the package oncoPredict [23] in R. In processing the dataset samples, we leveraged the data from 198 drugs available in the Genomics of Drug Sensitivity in Cancer (GDSC2) database (https://www.cancerrxgene.org/) for sample data. For patients, the half-maximal inhibitory concentration (IC50) values for each drug were calculated. Subsequently, to find drugs with significant correlations to biomarker expression levels, we applied the Spearman correlation coefficient analysis method and imposed stringent screening criteria (|cor| ≥ 0.3, *p* < 0.01).

### 2.6. Target Drug Prediction and Molecular Docking

To explore the regulatory mechanisms of *SNHG25* through the competing endogenous RNA (ceRNA) network in COAD and identify potential therapeutic target drugs, we first utilized the Encori database (https://rnasysu.com/encori/) to mine mRNA molecules that interact with *SNHG25*. Subsequently, we conducted GSEA on these mRNAs using the Enrichr package [24] in R and using the DSigDB database (https://dsigdb.tanlab.org/DSigDBv1.0/) to predict drug targets for the identified biomarkers. In the further analysis process, we screened receptor protein crystal structures related to these biomarkers through the UniProt database (https://www.uniprot.org/). During screening, we prioritized structures obtained by x-ray technology to ensure data accuracy and reliability. Furthermore, we chose structures with higher Ångstroms, which is a commonly used unit of measurement for light wavelength and molecular diameter. Higher Ångstrom indicated a finer structure, which can more accurately reflect the binding sites of proteins. We also considered the length of protein chains, preferring longer ones that potentially contain more binding sites. In cases where single-chain proteins were available, we opted for them to simplify subsequent analysis and docking procedures. Based on the predictions from DSigDB, we downloaded the corresponding 3D structures of targeted drugs from the PubChem website (https://pubchem.ncbi.nlm.nih.gov/) as ligands. For drugs lacking 3D structures or requiring specific gene overexpression or knockout conditions, we made reasonable alternative selections based on *p* value rankings. Next, we used PyMOL software to perform meticulous preprocessing on all molecules, including the removal of water molecules, addition of hydrogen atoms, and elimination of small molecule impurities, to ensure a pure docking environment. Subsequently, we employed ChemBioOffice software to minimize the energy of the 3D structures of the targeted drugs, thereby optimizing their conformations and augmenting docking precision. Ultimately, AutoDocktools was utilized to execute docking simulations between receptor proteins and drug molecules, which were then rigorously screened against predetermined criteria. These criteria included a binding energy of less than −5 kcal/mol to ensure stable binding between the drug and receptor and a hydrogen bond length limited to within 3.5 Å to maintain the specificity of molecular interactions.

### 2.7. Cell Culture and Generation of Stable Knockdown COAD Cell Lines

Human normal colon epithelial cell lines (NCM460) and colorectal cancer cell lines (HCT116) were sourced from the American Type Culture Collection (ATCC, Manassas, MD, Uuited States). The LoVo colorectal cancer cell lines were acquired from Beyotime (Shanghai, China). Both NCM460 and HCT116 cells were grown in RPMI-1640 medium enriched with 10% fetal bovine serum (FBS, Hyclone, Logan, UT, United States), while LoVo cells were maintained in DMEM medium supplemented with 10% FBS and antibiotics (penicillin at 100 U/mL and streptomycin at 0.1 mg/mL). All cell lines were incubated at 37°C in an atmosphere of 5% CO_2_.

To achieve the knockdown of *SNHG25*, HCT116 and LoVo cells were transfected using a lentiviral vector that encodes shRNA specifically targeting *SNHG25* (5⁣′-3⁣′: GGATGTCATCGTCCTTGCT, designated as sh-*SNHG25*#1; 5⁣′-3⁣′: CCCGTCAATAAAGTGGTTTGA, referred to as sh-*SNHG25*#2) or a control vector lacking the shRNA (sh-NC) (provided by Genepharma, Shanghai, China) in the presence of polybrene at a concentration of 5.0 *μ*g/mL. After 24 h postinfection, the cells were subjected to selection with puromycin at a concentration of 0.2 mg/mL. The efficiency of *SNHG25* knockdown was validated through quantitative real-time polymerase chain reaction (qRT-PCR).

### 2.8. RNA Extraction and qRT-PCR

RNA was isolated from cell cultures utilizing TRIzol reagent (Life Technologies, United States). The concentration of RNA was determined with a NanoDrop ND-2000 spectrophotometer (NanoDrop Technologies, Wilmington, DE, United States). Reverse transcription of RNA to cDNA was carried out using a reverse transcription kit (Takara, Tokyo, Japan). qRT-PCR was conducted with the SYBR qPCR Master Mix (Vazyme, Nanjing, China), following the guidelines provided by the manufacturer. The sequences of the primers were SNHG25 forward primer 5⁣′-GCAGGTTCCGGGAGGTCA-3⁣′, SNHG25 reverse primer 5⁣′-CAAACCACTTTATTGACGGGAA-3⁣′, glyceraldehyde-3-phosphate dehydrogenase (GAPDH) forward primer 5⁣′-AGAAGGCTGGGGCTCATTTG-3⁣′, and GAPDH reverse primer 5⁣′-AGGGGCCATCCACAGTCTTC-3⁣′.

The parameters employed included an initial predenaturation step at 95°C for 10 min, followed by 40 cycles of denaturation at 95°C for 30 s, annealing at 60°C for 1 min, and extension at 60°C for 30 s. The housekeeping gene GAPDH served as the internal control. The 2^−ΔΔCT^ method was utilized to calculate the fold change in the expression levels of the target genes.

### 2.9. Cell Proliferation, Migration, and Invasion Assays

Cell proliferation was evaluated utilizing the cell counting kit-8 (CCK-8; MedChemExpress, Monmouth Junction, NJ, United States), in accordance with the guidelines provided by the manufacturer. HCT116 and LoVo cells in the logarithmic growth phase that had undergone transfection were plated in triplicate in a 96-well plate, with a density of 5000 cells per well. They were then incubated for 24 h at 37°C in an atmosphere containing 5% CO_2_. Following this, the cells were treated with 100 *μ*L of culture medium and 10 *μ*L of CCK-8 for 1 h. The absorbance readings for each well were taken at 450 nm at four time points—0, 24, 48, and 72 h—using a microplate reader (Tecan, Mechelen, Belgium).

The scratch assay was utilized to investigate cell migration. Cells that had been transfected were plated in triplicate in two 6-well plates containing complete medium and allowed to grow until they reached 100% confluence. Once confluence was achieved, a sterile pipette tip was used to create scratches in the plates. The medium was then replaced with serum-free RPMI-1640, and images were captured under a microscope at 0 and 48 h. The width of the scratch was monitored over time with ImageJ software to assess cell migration.

An invasion assay of COAD cells was conducted utilizing a transwell chamber (Millipore, Billerica, MA, United States) that was precoated with a Matrigel basement membrane matrix (BD Biosciences, Franklin Lakes, NJ, United States). A volume of 200 *μ*L containing 4 × 10^4^ transfected cells suspended in RPMI-1640 medium without serum was introduced into the upper chamber lined with Matrigel. The lower chamber received 600 *μ*L of RPMI-1640 medium enriched with 10% FBS. The incubator was set to 37°C, and the setup was left undisturbed for 36 h. Afterward, cells that migrated through the 8 *μ*m membrane were fixed and stained utilizing crystal violet. An inverted microscope was employed to count the number of cells in three randomly selected fields.

### 2.10. Statistical Analysis

All the statistical analyses were performed in R language (Version 3.6.0) or GraphPad Prism software (version 8.0.2). The Wilcoxon rank-sum test was employed to calculate the difference between two groups of continuous variables. Spearman's correlation analysis was used to compute correlations. Multiple group comparisons in cellular experiments were performed using Student's *t*-test or one-way and two-way analysis of variance (ANOVA). All experimental data were concluded from three independent replicated tests. *p* < 0.05 denoted statistically significant.

## 3. Results

### 3.1. Screening of DEGs and Expression of *SNHG25*

**Figure 1:**
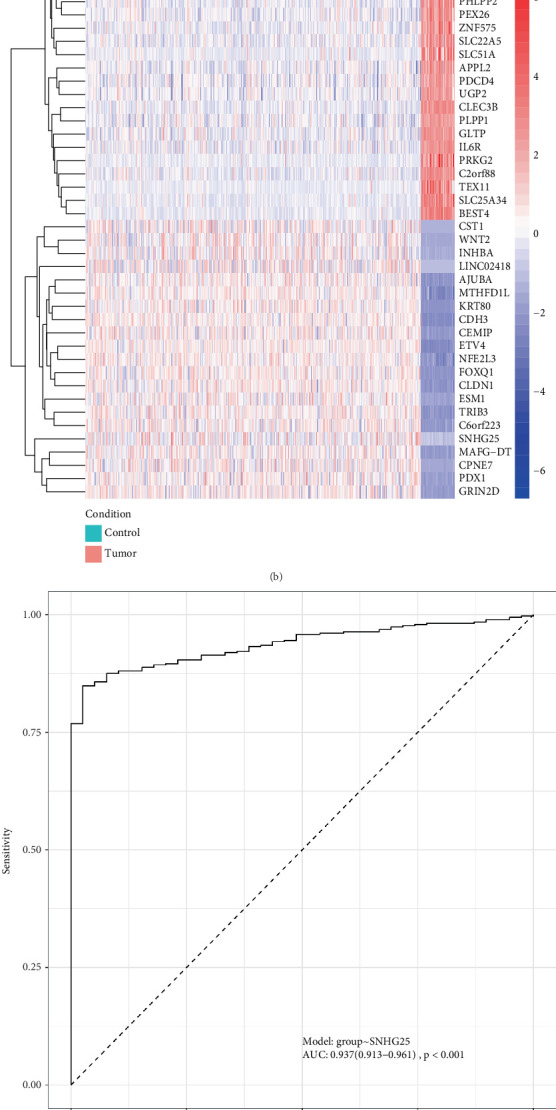
Screening of DEGs in COAD and the expression of *SNHG25*. (a) The volcano plot for DEGs between the control group and the COAD group, where the horizontal axis represents log2FC in differential expression, and the vertical axis represents −log10(adj.*p*.*v*al). Each dot in the plot represents a gene, with blue dots in the left half representing downregulated genes and red dots in the right half representing upregulated genes. (b) The heat map showcasing the expression levels of the Top 20 genes with the highest and lowest significance of differential expression, along with the *SNHG25* gene, where red represents high expression and blue represents low expression. (c) The ROC curve for *SNHG25*, with an AUC greater than 0.9, demonstrates the gene's exceptionally strong predictive power. (d) A boxplot showcasing the expression levels of *SNHG25*, with blue representing the COAD group and red representing the control group.

After conducting an in-depth analysis of gene expression data between tumor and control groups using the DESeq2 package [16], we successfully identified and screened out 8666 significantly DEGs, comprising 4804 upregulated genes and 3862 downregulated genes (Figure 1a). Notably, the lncRNA *SNHG25* emerged as one of the significantly upregulated genes (log2FC = 4.65, *p* < 0.01). To visually illustrate the expression patterns of these key genes, the Top 20 genes with the highest and lowest significance of differential expression were selected, incorporating *SNHG25* specifically, totaling 41 genes, and plotted a heat map of their expression levels (Figure 1b). Furthermore, we generated an ROC curve for *SNHG25* and calculated its area under curve (AUC) value, which was 0.937 (95% CI: 0.913–0.961). An AUC value greater than 0.7 is generally considered to indicate good diagnostic performance, indicating that *SNHG25* exhibits extremely high accuracy and specificity in distinguishing COAD patients from healthy individuals (Figure 1c). Additionally, we visually presented the expression difference of *SNHG25* between the COAD group and the control group through a boxplot (Figure 1d). Similarly, we found in the GSE146009 dataset that the expression level of *SNHG25* was significantly upregulated in COAD samples (Figure S1, *p* = 0.0014). The results showed that the expression level of *SNHG25* in the COAD group was remarkably higher than that in the control group (*p* < 0.01), reinforcing the reliability of *SNHG25* as a DEG.

### 3.2. The Correlation Between *SNHG25* and Apoptosis in COAD

**Figure 2:**
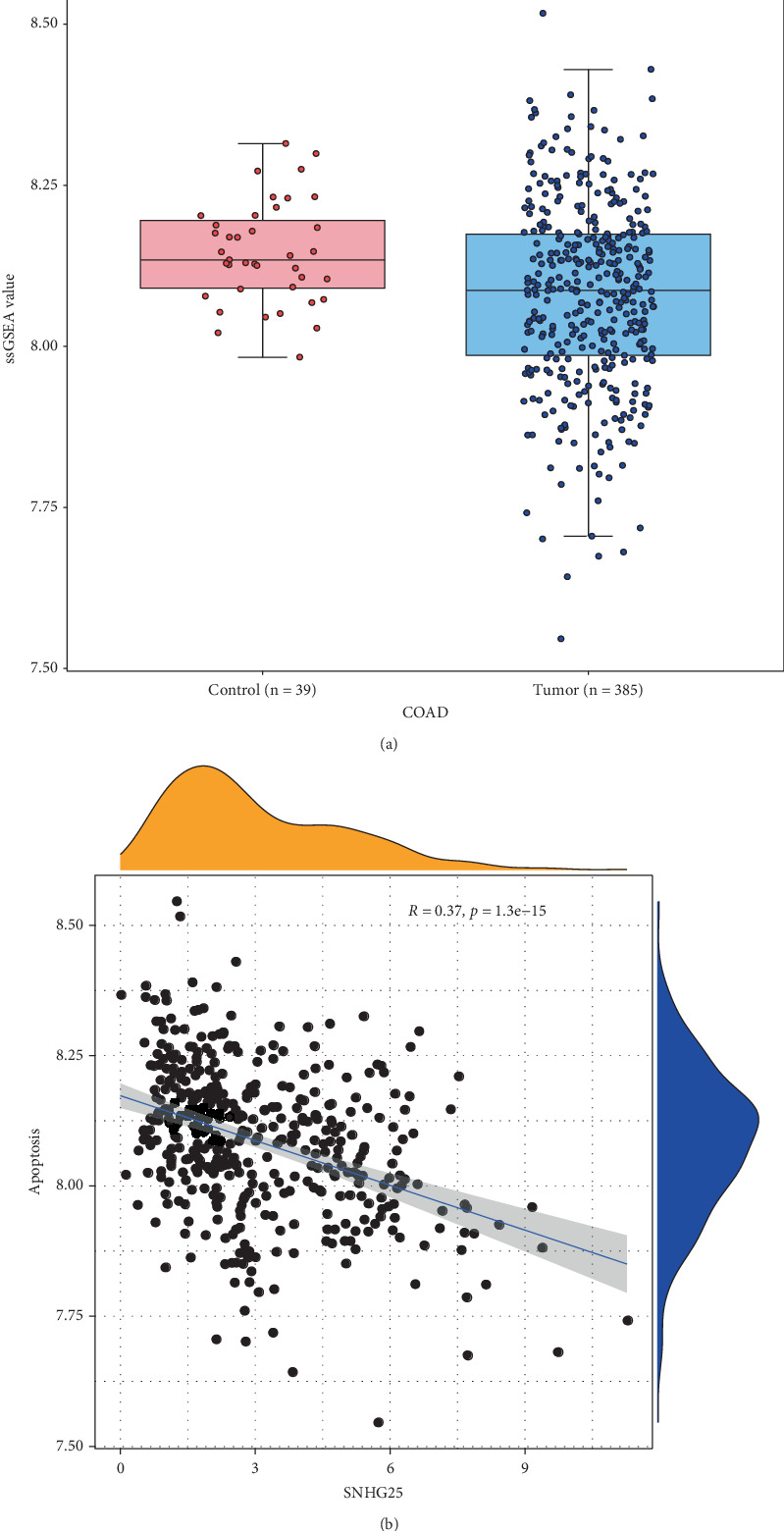
Association of *SNHG25* with apoptosis in COAD. (a) Boxplot of apoptosis scores between tumor samples and control samples, with blue representing the COAD group and red representing the control group. (b) Spearman's correlation plot between *SNHG25* expression and apoptosis score.

Using apoptosis-related genes as the background gene set, we calculated the PDS for samples. It was found that the PDS in control samples was notably higher than that in cancer samples (*p* < 0.01) (Figure 2a). Subsequently, a Spearman correlation analysis was performed between the expression levels of *SNHG25* and the apoptosis-related score, revealing a significant negative relation between *SNHG25* and apoptosis (*R* = −0.37, *p* < 0.01, Figure 2b). This finding is in line with previous research outcomes [7].

### 3.3. GSEA of *SNHG25*

**Figure 3:**
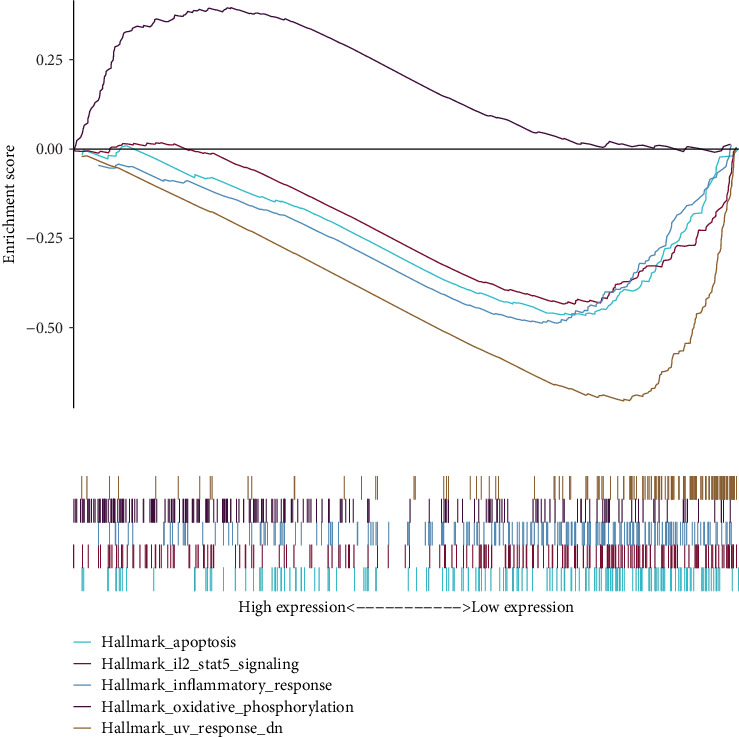
GSEA results of the high- and low-expression groups of SNHG25. As we move from left to right, an ES value is calculated for each gene, and these values are connected to form a line. The peaks and troughs formed represent the ES values of the gene set phenotype. A positive ES indicates that a functional gene set is enriched towards the front of the ranked list, whereas a negative ES indicates that a functional gene set is enriched towards the end of the ranked list. The lower part represents the distribution of gene expression levels within that particular pathway.

Enrichment analysis revealed that high *SNHG25* expression was associated with oxidative phosphorylation, indicating enhanced mitochondrial metabolism that supports tumor growth and survival. In contrast, low *SNHG25* expression enriched apoptosis, inflammatory response, and IL2-STAT5 signaling, suggesting potential tumor-suppressive effects via cell death and immune activation. These results suggest that *SNHG25* may contribute to the onset and progression of COAD by promoting either a metabolic reprogramming approach or immune escape, which in turn drives COAD (Figure 3 and Table S1).

### 3.4. Correlation Between *SNHG25* and Immune Infiltration

**Figure 4:**
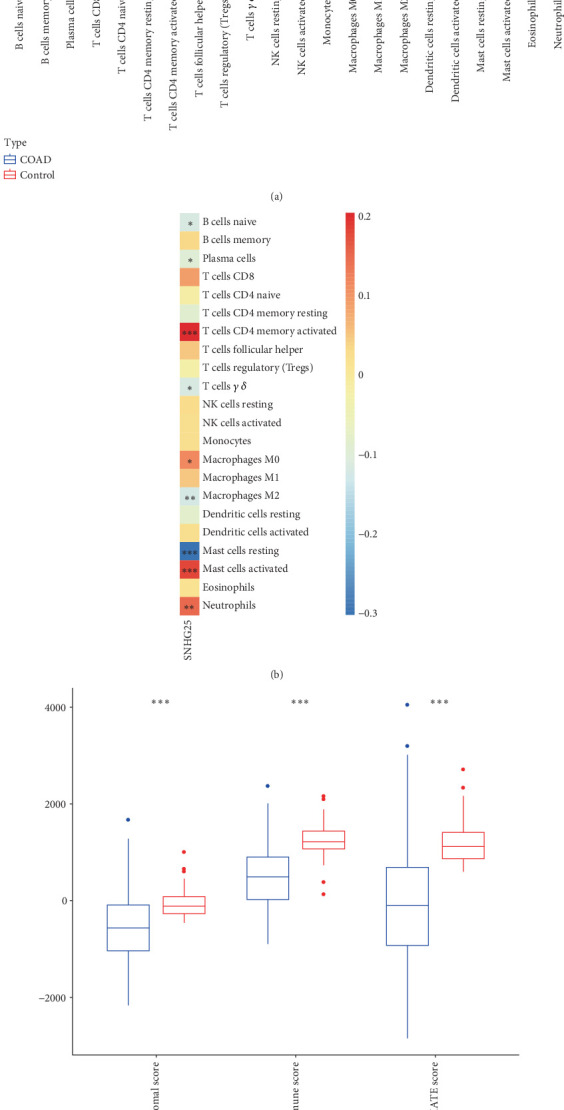
Infiltration of *SNHG25* immune cells and its correlation analysis. (a) Boxplot showcasing the infiltration levels of 22 types of immune cells as determined by CIBERSORT. (b) Spearman's correlation analysis diagram between *SNHG25* and 22 types of immune cell infiltration. (c) The immune score, stromal score, and ESTIMATE score of the control group and COAD group obtained by the ESTIMATE algorithm. (d) Spearman's correlation analysis diagram between *SNHG25* and stromal infiltration score, immune score, and ESTIMATE score. ⁣^∗^*p* < 0.05; ⁣^∗∗^*p* < 0.01; ⁣^∗∗∗^*p* < 0.001.

We conducted an immune infiltration analysis on our samples, revealing significant differences (*p* < 0.05, Figure 4a) in the infiltration patterns of 16 immune cell types between tumor and control groups, including plasma cells, T cells, macrophages, and NK cells. Upon calculating the correlation coefficients between *SNHG25* expression and immune cell infiltration scores, we found that the infiltration status of nine immune cells, including macrophages M0, macrophages M2, B cells naive, plasma cells, T cells gamma delta, mast cells resting, T cells CD4 memory activated, neutrophils, and mast cells activated, were significantly correlated with *SNHG25* expression levels (*p* < 0.05, Figure 4b) and discovered that the immune score, stromal score, and ESTIMATE score were all significantly higher in the control group compared to the tumor samples (*p* < 0.05, Figure 4c). Furthermore, these scores were all significantly negatively correlated with *SNHG25* expression levels (*p* < 0.01, Figure 4d). This finding suggests a potential role for *SNHG25* in shaping the TME, particularly in inhibiting immune cell infiltration and stromal remodeling.

**Figure 5:**
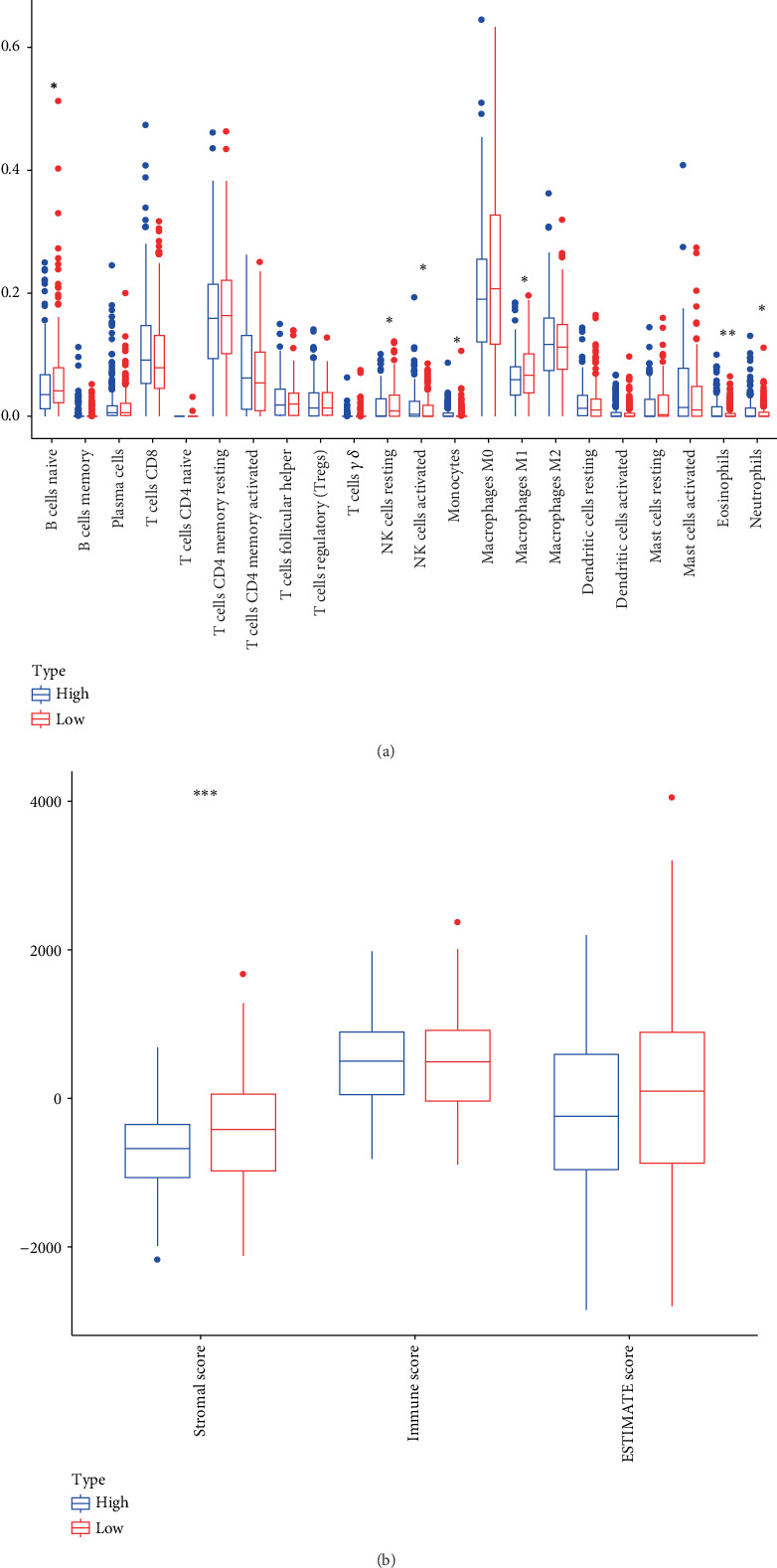
Comparison of immune cell infiltration between high- and low-expression groups. (a) Boxplot of the infiltration of 22 immune cell types in high- and low-expression groups. (b) Boxplot of the ESTIMATE immune infiltration in high- and low-expression groups. ⁣^∗∗∗^*p* < 0.001.

The analysis of immune cell infiltration differences between high-expression and low-expression groups revealed that the expressions of neutrophils, NK cell activated, mast cell activated, and eosinophils were higher in the high-expression group compared to the low-expression group, whereas the low-expression group exhibited higher expression of B cells naive, NK cells resting, and macrophages M1 than the high-expression group (*p* < 0.05, Figure 5a). Compared to the low-expression group, stromal score was significantly lower in the high-expression group (*p* < 0.001, Figure 5b).

### 3.5. Correlation Analysis of *SNHG25* With Immunotherapy and Drug Sensitivity

**Figure 6:**
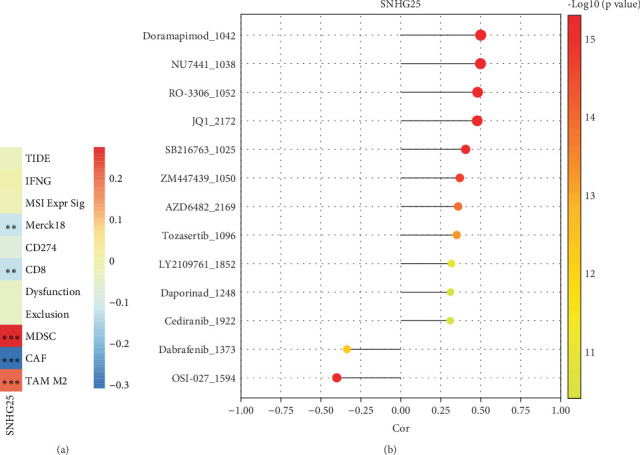
Correlation analysis of *SNHG25* with immunotherapy and drug sensitivity. (a) Correlation analysis between *SNHG25* and immune cell marker scores. (b) Lollipop plot of the correlation between *SNHG25* and the 13 drugs. ⁣^∗∗^*p* < 0.01; ⁣^∗∗∗^*p* < 0.001.

The association between *SNHG25* expression levels and various immune cell markers in the samples was explored to evaluate the potential clinical effects of immunotherapy within the TIDE database. The results indicated that *SNHG25* expression was significantly negatively related to the expression of Merck18, CD8, and CAF, while it showed a significant positive relation with the expressions of myeloid-derived suppressor cell (MDSC) and tumor-associated macrophage (TAM) M2 (Figure 6a, *p* < 0.01).

To further explore the therapeutic significance of *SNHG25* as a potential biomarker, we calculated IC50 values for these drugs in patients, and we have successfully identified 13 drugs that exhibit heightened sensitivity to variations in *SNHG25* expression levels (Figure 6b, |cor| ≥ 0.3, *p* < 0.01). Among them, dabrafenib_1373 and osii-027_1594 are two drugs that inhibit the expression of SNHG25, while the remaining 11 drugs enhance the expression of *SNHG25* (Figure 6b, *p* < 0.01).

### 3.6. Prediction and Molecular Docking of *SNHG25*-Regulated mRNA Drugs

**Figure 7:**
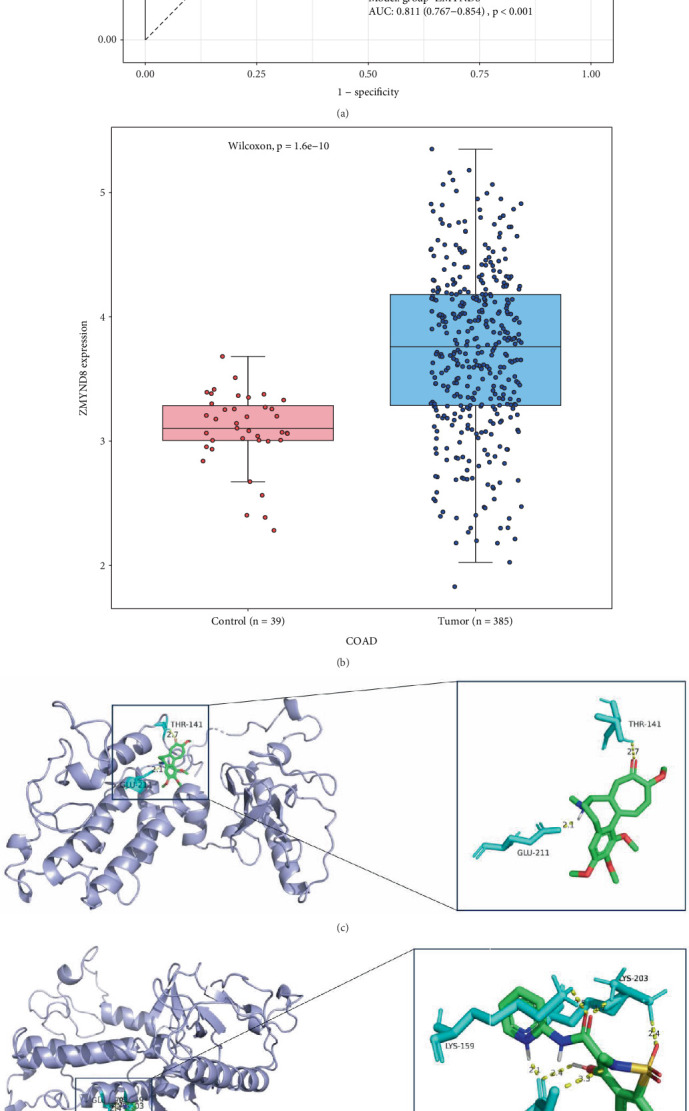
Identification of the key gene *ZMYND8* regulated by *SNHG25* and results of molecular docking and drug prediction based on *ZMYND8*. (a) The ROC curve for *ZMYND8*, with an AUC greater than 0.8, demonstrates the gene's exceptionally strong predictive power. (b) A boxplot showcasing the expression levels of *ZMYND8*, with blue representing the COAD group and red representing the control group. Molecular docking results of *ZMYND8* with (c) demecolcine, (d) piroxicam, and (e) vorinostat. The green molecule represents the small drug molecule. The blue molecule represents the receptor protein, and the cyan color represents amino acids. The hydrogen bonds between the receptor protein and the small drug molecule are indicated by yellow dashed lines, with the numbers above indicating the hydrogen bond lengths in Ångstrom.

In order to explore the molecular mechanisms of COAD, we utilized Encori to inquire into the mRNAs regulated by *SNHG25* via the ceRNA network in COAD, uncovering zinc finger MYND-type containing 8 (*ZMYND8*) as a pivotal mRNA. Specifically, our research reveals that *ZMYND8* exhibits remarkable diagnostic performance in COAD, with an AUC value of 0.811 (95% CI: 0.913–0.961, *p* < 0.001), and this gene displays a notably upregulated expression trend in COAD samples, aligning with the characteristics of the ceRNA network (Figure 7).

Having introduced *ZMYND8* into the Enrichr package [24], we predicted potential drugs targeting this biomarker within the DSigDB database. Only drugs with significant enrichment (*p* < 0.05), available 3D structural data in PubChem, and potential relevance to tumor-related processes (e.g., apoptosis induction and antiangiogenesis) were prioritized for molecular docking analysis. After rigorous screening, we selected demecolcine, piroxicam, and vorinostat as the candidates for our molecular docking analysis. To assess their interactions with ZMYND8, we chose the 4COS structure of ZMYND8 as the protein target for docking. The docking site was placed within a cubic box centered on the initial ligand. It is important to note that a lower docking score (more negative value) indicates a higher binding affinity between the compound and the protein. The docking results demonstrated that all three drugs exhibited strong binding affinities towards the ZMYND8 protein, as shown by their binding energies significantly lower than the −5.0 kcal/mol threshold. Specifically, the binding affinities of demecolcine, piroxicam, and vorinostat were −6.48, −7.15, and -5.39 kcal/mol, respectively (Tables 1 and 2). A visual analysis of docking of the 4COS protein (Figures 7c, 7d, and 7e) showed that all three drugs formed stable conformations with the protein through hydrogen bonding interactions.

### 3.7. *SNHG25* Knockdown Inhibits Proliferation, Migration, and Invasion of COAD Cells

**Figure 8:**
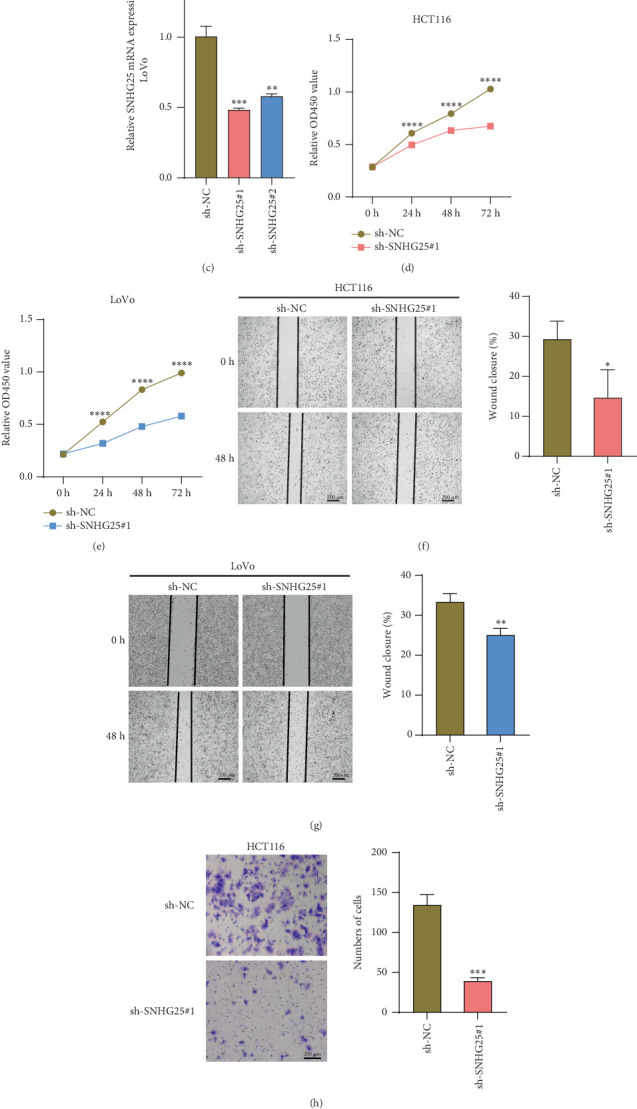
In vitro cellular assay to validate the expression and potential biological functions of *SNHG25* in COAD cells. (a) Using qRT PCR to validate the mRNA expression levels of *SNHG25* in NCM460, HCT116, and LoVo cells. Validation of knockdown efficiency of *SNHG25* in (b) HCT116 and (c) LoVo cells, respectively. CCK-8-based assay to validate the effect of *SNHG25* knockdown on the proliferation levels of (d) HCT116 and (e) LoVo cells. Based on a wound healing assay to validate the effect of *SNHG25* knockdown on the migration levels of (f) HCT116 and (g) LoVo cells. Based on a wound healing assay to validate the effect of *SNHG25* knockdown on the migration levels of (h) HCT116 and (i) LoVo cells. ⁣^∗^*p* < 0.05, ⁣^∗∗∗^*p* < 0.01, ⁣^∗∗^*p* < 0.001, and ⁣^∗∗∗∗^*p* < 0.0001.

To further validate the functional role of *SNHG25* in COAD, we constructed *SNHG25* knockdown models in HCT116 and LoVo cell lines, respectively. qRT-PCR results showed that the expression of *SNHG25* was significantly higher than that in normal intestinal epithelial cells in COAD cells (Figure 8a), and the shRNA intervention significantly reduced its expression level (Figure 8b,c). The CCK-8 assay showed that *SNHG25* knockdown significantly inhibited the proliferative ability of both cell lines within 72 h (Figure 8d,e). Further, it was observed by wound healing assay that the migratory ability of HCT116 with LoVo cells was significantly decreased after *SNHG25* knockdown (Figure 8f,g). The transwell assay also verified that the invasive ability of cells in the *SNHG25* knockdown group was significantly lower than that in the control group (Figure 8h,i). These results indicate that *SNHG25* has proproliferative, promigratory, and proinvasive functions in COAD cells, suggesting its potential oncogenic role in COAD.

## 4. Discussion

COAD is a prevalent and deadly malignancy worldwide [25]. Previous studies have found that overexpressed *LINC00152* can promote the expression of fascin actin-bundling protein 1 (*FSCN1*) by sponging *miR-632* and *miR-185-3p*, leading to proliferation and metastasis in COAD [26], suggesting that lncRNA *SNHG25* in this study may also affect the occurrence of COAD tumor cells by influencing microRNA (miRNA). *SNHG25*, a member of the lncRNA family, has been proven to promote the proliferation and migration of cancer cells in COAD [7]. Against this backdrop, the present study, leveraging databases, acquired datasets comprising cancer samples and adjacent normal tissues. Through a range of methods including differential analysis, correlation analysis, enrichment analysis, and immune infiltration analysis, we delved into the relationship between *SNHG25* and the onset of COAD. Notably, we reveal a possible underlying mechanism whereby the expression level of *ZMYND8* may be altered upon binding to specific antitumor drugs, which in turn regulates the availability of miRNAs through the ceRNA mechanism and indirectly downregulates the expression of *SNHG25*, thereby inhibiting the progression of COAD (Figure S2).

The *SNHG25*, located at 17q23 on the human chromosome, is a crucial lncRNA whose molecular mechanisms of action have yet to be fully elucidated [7]. Regarding its relationship with cancer, studies have already demonstrated that *SNHG25* positively regulates the upregulation of MAP2K2 in glioma cells and tissues by sponging *miR-579-5p* [27]. Previous studies have also found that *SNHG25* can promote the malignancy of endometrial cancer through miRNA sponges [12], and it can affect the invasion and apoptosis of renal cancer cells by regulating the miR-363-3p-Twist1 interaction [28]. Gong et al. discovered that PAX 5 can activate the transcription of *SNHG25* in CRC cells [7]. This study found that different expression levels of *SNHG25* lead to distinct enriched pathways. High expression of *SNHG25* results in significant enrichment of the oxidative phosphorylation pathway. Recent studies have revealed that oxidative phosphorylation not only provides sufficient energy for the survival of tumor tissues but also regulates tumor proliferation, invasion, and metastasis [29]. In contrast, low expression of *SNHG25* leads to significant enrichment of signaling pathways such as apoptosis, inflammatory response, and IL2/STAT5. Among them, apoptosis is an important mechanism for suppressing cancer, and the inflammatory response is a normal defense mechanism of the body, which helps to eliminate abnormal cells and pathogens. Moreover, the inflammatory response mediated by macrophages and NK cells can participate in the surveillance and clearance of tumor cells [30]. Activation of the IL2/STAT5 pathway can enhance the antitumor function of CD8^+^ T cells [31]. These results suggest that SNHG25 may play an important role in regulating metabolism and the immune microenvironment in COAD.

An analysis of the correlation between *SNHG25* and immune cell infiltration has uncovered a close association between the expression of this lncRNA and nine core immune cell types. Additionally, an examination of immune cell markers has shown that MDSCs and the M2 subtype of TAMs can notably enhance the expression of *SNHG25*. It is widely recognized that various immune cell types, including T cells, neutrophils, macrophages, NK cells, dendritic cells, and B cells, exist in the TME and play a pivotal role in cancer biology [32, 33]. Recent evidence indicates that lncRNA can regulate the differentiation and function of immune cells, such as dendritic cell activity, T cell ratio, and metabolism [34], and therefore represents a potential target for cancer therapy [35]. In innate immune cells, studies showed that lncRNAs could directly regulate some biological functions, including cytokine production, cell survival, and development. It has been reported that some lncRNAs are associated with the homeostasis of immune cells in vivo [36]. For instance, lncRNA *Morrbid* controls the homeostasis of Ly6C^hi^ monocytes, neutrophils, and eosinophils in the body by regulating *BCL2L11*, a proapoptotic molecule [37]. Some lncRNAs can serve as diagnostic markers and even targets for immunotherapy [38]. In summary, these findings not only strengthen the close connection between immune cells and the initiation of various tumors and their development, including COAD, but also suggest that lncRNA *SNHG25* may participate in the remodeling and regulation of the TME by affecting the function and distribution of these immune cells.

In this study, *ZMYND8*, a multifunctional transcription factor, was selected as a potential key mRNA drug target [39]. *ZMYND8*, recognized as a core component of various transcriptional regulatory complexes, has demonstrated significant tumor-suppressive effects in breast cancer [40] and prostate cancer [41] in previous studies, providing evidence for its potential as a biomarker candidate in this research. Given that the upregulation of *SNHG25* significantly enhances cancer cell invasiveness, two drugs, dabrafenib_1373 and OSI-027_1594, which exhibit significant negative correlations with *SNHG25* expression, were screened as therapeutic candidates. Dabrafenib, an efficient BRAF kinase ATP-competitive inhibitor, acts on the MAPK signaling pathway [42]. Its IC50 value against BRAFV600E in vitro is as low as 0.65 nM, and its effectiveness has been validated in clinical trials for thyroid cancer [43] and non–small cell lung cancer [44]. On the other hand, OSI-027, a dual inhibitor of mechanistic target of rapamycin complex 1/2 (mTORC 1/2), has shown potential activity against pancreatic cancer cells both in vitro and in vivo, inducing apoptosis in these cells [45]. Furthermore, this study discovered three potential drugs: demecolcine, piroxicam, and vorinostat. Demecolcine is an effective mitotic inhibitor that has been previously studied as a potential drug for colon cancer [46]. Piroxicam, a significant anti-inflammatory drug, is a primary candidate for chemoprevention in inflammatory bowel disease patients. It downregulates redox sensitivity and inflammatory transcription factors NF*κ*B and AP-1, reducing their DNA-binding capabilities [47]. Animal studies in colon cancer have confirmed that piroxicam can decrease tumor incidence and size [48, 49]. Vorinostat (suberoylanilide hydroxamic acid [SAHA]), an inhibitor of histone deacetylase Classes I and II, is an effective anticancer drug [50]. Clinical trials have demonstrated its crucial role in antiangiogenic therapies, particularly in patients with metastatic sarcomas or metastatic CRC, corroborating the predictions of this study [51]. Our analysis supports the potential therapeutic value of these five drugs for COAD, though comprehensive assessment of their efficacy still necessitates rigorous in vitro and in vivo experimental data.

Although this study successfully uncovered the association between the *SNHG25* and COAD, and preliminarily identified potential drugs based on this finding, it is accompanied by several limitations. First, this study mainly relied on data from public databases for analysis, and although the sample size was representative, it still suffered from population bias, sample heterogeneity, and lacked further validation in a multicenter clinical cohort. In the future, we will combine independent cohorts from multiple regions and platforms for validation analysis and introduce real-world samples for retrospective or prospective studies to improve the broad applicability and stability of the results. Second, this study proposed a hypothetical pathway for *ZMYND8* to regulate *SNHG25* through a ceRNA mechanism, but the mechanism has not been confirmed by experiments at the molecular or animal level and is somewhat speculative. We will carry out molecular biology methods such as dual luciferase reporter assay and miRNA intervention assay in the follow-up study to further verify the authenticity and functionality of the *ZMYND8*-miRNA-*SNHG25* regulatory axis. Third, although we predicted the binding potential of *ZMYND8* with the three drugs using molecular docking, we did not conduct subsequent drug treatment experiments to observe the effects on *SNHG25* expression and cell behavior. Therefore, further drug treatment experiments were conducted to assess the effects of specific drugs on *ZMYND8*/*SNHG25* expression and colon cancer cell proliferation, migration, and immune response.

## 5. Conclusion

This study, leveraging bioinformatics techniques, explores the intricate relationship between the lncRNA *SNHG25* and COAD. Based on the crucial mRNA *ZMYND8* regulated by *SNHG25*, we proposed three potential drug candidates: demecolcine, piroxicam, and vorinostat. This research underscores the pivotal role of lncRNA *SNHG25* as a potential biomarker for COAD, offering fresh perspectives and potential avenues for COAD diagnosis, prognosis evaluation, and targeted therapy. This discovery not only enriches our understanding of the complex pathological process of COAD but also emphasizes the significance of lncRNA in cancer occurrence and progression, providing a new perspective for research on cancer.

## Figures and Tables

**Table 1:** Binding energies of molecular docking between three drugs and 4COS.

**Compound CID**	**Molecular_name**	**Gene_name**	**PDB_ID**	**Energy (kcal/mol)**
220401	Demecolcine	ZMYND8	4COS	−6.48
54676228	Piroxicam	ZMYND8	4COS	−7.15
5311	Vorinostat	ZMYND8	4COS	−5.39

**Table 2:** Molecular docking parameters of ZMYND8 binding to three drugs.

**Term**	**Spacing**	**Npts**	**Center**
ZMYND8_demecolcine	0.614	126, 126, 126	41.775, 35.839, 5.055
ZMYND8_piroxicam	0.492	126, 126, 126	41.775, 35.839, 5.055
ZMYND8_vorinostat	0.514	126, 126, 126	41.775, 35.839, 5.055

## Data Availability

The data that support the findings of this study are available from the corresponding author upon reasonable request.

## Nomenclature

COAD: colon adenocarcinoma
CRC: colorectal cancer
ncRNA: noncoding ribonucleic acid
lncRNA: long noncoding ribonucleic acid
DEG: differentially expressed gene
ROC: receiver operating characteristic
FDR: false discovery rate
GSEA: gene set enrichment analysis
KEGG: Kyoto Encyclopedia of Genes and Genomes
GO: Gene Ontology
GDSC: Genomics of Drug Sensitivity in Cancer
IC50: half-maximal inhibitory concentration
AUC: area under curve
PDS: programmed death score
TME: tumor microenvironment
ceRNA: competing endogenous ribonucleic acid
ES: enrichment score
NK: natural killer
NLR: neutrophil lymphocyte ratio
TAM: tumor-associated macrophage
MDSC: myeloid-derived suppressor cells
ZMYND8: zinc finger MYND-type containing 8
MAPK: mitogen-activated protein kinase
mTORC1/2: mechanistic target of rapamycin complex 1/2
Å: Ångstrom
SAHA: suberoylanilide hydroxamic acid
EMT: epithelial–mesenchymal transition
ESTIMATE: Estimation of STromal and Immune cells in MAlignant Tumor tissues using Expression data
miRNAs: microRNAs
NSCLC: non–small cell lung carcinoma
FSCN1: fascin actin-bundling protein 1

## Supporting Information

Supporting Information Additional supporting information can be found online in the Supporting Information section. Figure S1: Based on the GSE146009 dataset to analyze the difference in expression levels of SNHG25 in COAD and control samples. Figure S2: Schematic representation of the potential mechanism of action of SNHG25 in this study. Table S1: Enrichment information about the apoptotic pathway and the genes included in this pathway.
