# Supplementary material for: Bioinformatics Analysis Reveals the Oncogenic Role and Therapeutic Potential of lncRNA SNHG25 in Colon Adenocarcinoma
Source: Int J Genomics. 2025 Aug 23;2025:4528082. doi: 10.1155/ijog/4528082 (PMC12398412; doi:10.1155/ijog/4528082)
Supplement: Supporting Information — Additional supporting information can be found online in the Supporting Information section. Figure S1: Based on the GSE146009 dataset to analyze the difference in expression levels of SNHG25 in COAD and control samples. Figure S2: Schematic representation of the potential mechanism of action of SNHG25 in this study. Table S1: Enrichment information about the apoptotic pathway and the genes included in this pathway. [file 4528082.f1.zip › Supplementary Figures/Description of supplementary figures.docx]

**Figure S1.** Based on the GSE146009 dataset to analyze the difference in expression levels of SNHG25 in COAD and control samples.

**Figure S2.** Schematic representation of the potential mechanism of action of SNHG25 in this study.
